# Study on the effect of excavation sequence of three-hole shield tunnel on surface settlement and segment deformation

**DOI:** 10.1038/s41598-023-43936-8

**Published:** 2023-10-08

**Authors:** You Wang, Fang Dai, Bosong Ding, Ming Zhong, Heng Zhang

**Affiliations:** 1 School of Civil Engineering, Central South University, Changsha, 410075 China; 2 China Railway (Shanghai) Investment Group Co., Ltd., Shanghai, 200126 China; 3 China Railway ERJU 5Th Engineering Co., Ltd, Chengdu, 610091 China

**Keywords:** Civil engineering, Computational science

## Abstract

In order to study the influence of excavation sequence of three-hole parallel shield tunnel on surface settlement and segment convergence, an improved “three-stage analysis method” was proposed to calculate the surface settlement of three-hole parallel shield tunnel. Based on Peck's existing theory, this method deduced the ground settlement formula under the three-hole parallel condition, and can calculate the ground settlement more accurately. Based on the engineering background of a shield tunnel section in Jiangsu Province, a three-dimensional model of a three-hole parallel shield tunnel was established by using Flac3d software, and the three-hole parallel shield tunnel was simulated under four working conditions: right–center–left, right–left–center, right–left–right and right–center (reverse)–left. This paper analyzed the influence of tunnel excavation sequence on surface settlement, soil displacement and deformation of tunnel segments. The construction sequence was optimized based on the above influencing factors. It was found that Case 4, S-type sequential excavation, produced the least ground settlement. The surface settlement value caused by S-type excavation sequence was only 11.41 mm, and the convergence value of the segments generated by S-type excavation sequence was relatively small. Considering the economic factors such as construction efficiency and benefit, the S-shaped excavation sequence was better. The new calculation method of tunnel surface settlement and the optimal sequence of tunnel construction proposed in this paper can provide reference for actual construction.

## Introduction

Urban rail transportation is developing rapidly in the world today, and subways have become widely popular in most cities. Due to topography, planning and other restrictions, it is becoming more and more common to see double or even multiple tunnels constructed in parallel in existing examples of subway shield excavation projects. More and more related studies are also emerging. The ground settlement caused by double and multiple tunnels is also different from that of single tunnels. The same area of the ground surface will be subject to multiple disturbances, which will inevitably lead to more pronounced surface settlement, and therefore a study on the surface settlement of multiple parallel tunnels is needed.

For the calculation of surface settlement caused by single-hole tunnel excavation, Peck^1^ first proposed its shape as a normal distribution curve and proposed the classical Peck formula based on the concept of soil damage, after which many scholars made corrections on this basis. Zhu et al.^2^ considered the effects of grout filling rate, shield eccentricity ratio and support pressure ratio on tunnel surface settlement during shield excavation and established a modified Peck formula based on this. Xiang et al.^3^ introduced a correction factor for the width of the settlement slot using the excavation clearance parameters of shield tunnels. Based on this, the analytical solution of the peck formula for the settlement of shield tunnel construction was obtained, and the settlement trend analysis was carried out for the shallow buried single-hole tunnel. Combined with engineering calculations, the reliability of the proposed settlement prediction method was verified.

Existing studies are also more abundant for the calculation of surface settlement caused by tunneling in double-hole shields. Ma et al.^4^ extended the peck formula to a horizontal side-by-side double-hole tunnel, proposed a new double-hole settlement trough model, and verified the feasibility of the model. Kong et al.^5^ used the complex variable method to solve the analytical solutions of single-hole and double-hole tunnels. Based on this, the accuracy of the analytical solution for the double tunnel was verified based on the Schwartz alternating method. In addition, Kong et al. analytically investigated the effect of double tunnel distance on the surface settlement trough curve, which can provide guidance for the double tunnel design process. Franza et al.^6^ investigated the role of shear deformation capacity on the response of single- and double-hole tunnels. They implemented an equivalent Timucinco beam based on a soil-structure interaction model of the continuum for assessing the settlement and longitudinal deformation induced by excavation of existing tunnels and pipelines. Islam et al.^7^ summarized the surface settlement induced by twin-tunneling in the past decades. The study summarized the settlement induced by single-hole tunnels, volume loss, and factors that may affect the ground settlement induced by twin tunnels.

Due to the demand for urban development, the distribution of three-hole or even multi-holes tunnels is becoming common in addition to two-hole tunnels. Due to the demand of urban development, the distribution of three-hole and even multi-hole tunnels is gradually becoming common in addition to two-hole tunnels, but there are fewer studies on the ground settlement and tube sheet deformation in three-hole tunnels. Hu et al.^8^ explored the surface movement caused by three-level tunnels under different construction sequences. To this end, Hu et al. proposed an improved peck formula to predict the three-level tunnel-induced surface settlement and verified the validity of the proposed improved peck formula.

The current research and analysis methods on the surface deformation and ground settlement caused by shield tunnel boring of subway tunnels mainly include the empirical formula method^9,10^, theoretical analysis method^11,12^, numerical calculation method^13–15^, model test method^16–18^, artificial intelligence method^19–21^, field actual measurement method^22,23^. With the continuous progress of computing and simulation technology, simulation means have now started to be used in many fields such as civil construction, road, ground investigation and mining to analyze specific engineering problems. Numerical simulation can restore the actual engineering situation more realistically and help to solve engineering-related problems, which has become a commonly used technical tool. In the field of geotechnical engineering, the use of Flac3d can better simulate the real construction situation, reflecting the geological changes, specifically can be applied to the analysis of the tunneling process, slope stability, roadway support and other aspects.

Therefore, based on Peck's formula, this paper proposed the "three-stage analysis method" for the calculation of the settlement curve of three-hole parallel tunnels, and validate the theoretical analysis by combining field monitoring and numerical simulation. The effect of the spacing between the three tunnels on surface settlement and segment deformation is investigated. By combining theoretical equations, numerical simulations and field monitoring, the optimal construction sequence is obtained in order to guide the actual construction.

## Methodology

### Peck’s formula theory

**Figure 1:**
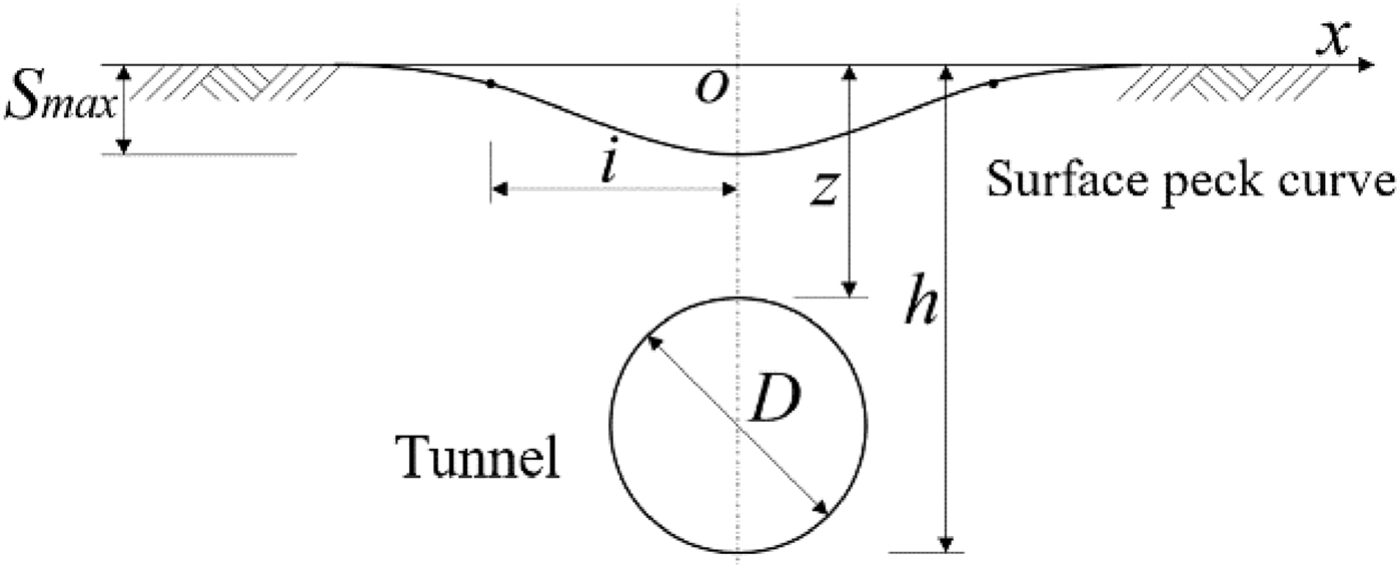
Schematic diagram of ground settlement for single-hole tunnel.

Since ground settlement induced by tunnel excavation will affect the ground surface and adjacent buildings and pipelines, it is crucial to predict ground settlement. A schematic diagram of the ground surface and deep soil settlement troughs induced by single-hole tunnel excavation is shown in Fig. 1.

Peck^1^ conducted research and analysis on the basis of a large amount of measured data, and found that the settlement characteristics of the strata in the lateral direction showed an approximate normal distribution curve, and proposed the following equation for surface settlement prediction:

1 $$ S\left( x \right) = S_{max} \cdot \exp \left[ {\frac{{ - x^{2} }}{{2i^{2} }}} \right] $$

2 $$ S_{{{\text{max}}}} = \frac{{V_{{{\text{loss}}}} }}{{i\sqrt {2\pi } }} = \frac{{\sqrt \pi D^{2} V_{L} }}{4\sqrt 2 \cdot i} $$

where S(x) denotes the vertical ground settlement at calculation point x (mm); x is the horizontal distance from calculation point to tunnel center (m); S_max_ is the maximum vertical ground settlement above the tunnel center point (mm); i represents the horizontal distance from the inverse bend point of Gaussian settlement curve to the tunnel center, i.e., the width coefficient of ground settlement trough (m); V_loss_ denotes the volume of soil loss; V_L_: soil loss rate; D: tunnel excavation diameter (m).

**Figure 2:**
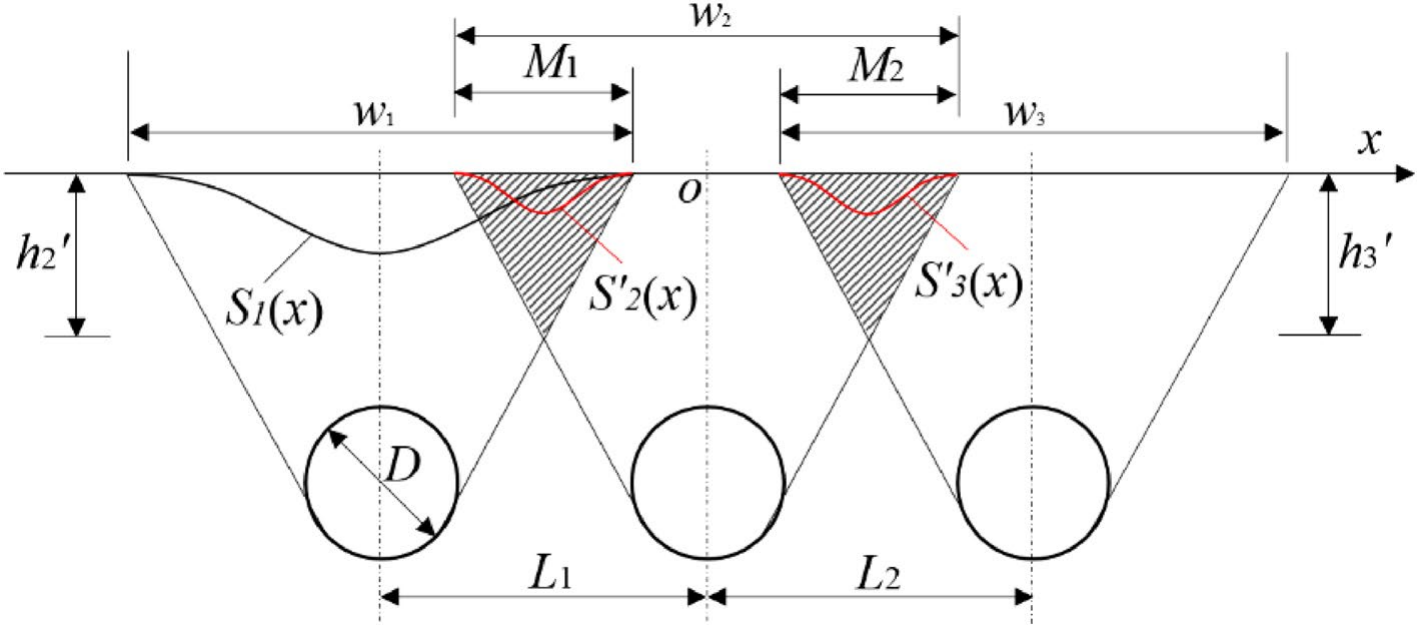
Schematic diagram of the repetitive disturbance area.

When the tunnel spacing is small, repeated disturbances will be generated for the surrounding rock, and the extent of the repeated disturbance area also directly determines the shape of the surface settlement distribution curve and the extent of settlement generated. Figure 2 shows the distribution of the repeated disturbance area formed after the excavation of the three tunnels. Where M is the width of the repeatedly disturbed area on the ground surface, W is the width of the disturbed area on the ground surface, h is the burial depth of the tunnel, and L is the spacing between the tunnel axes.

In this paper, the equations for W and M are given for three parallel tunnel excavations, as shown in Eqs. (3) and (4).

3 $$ W = \frac{{{\text{w}}_{1} }}{2} + \frac{{{\text{w}}_{3} }}{2} + L_{1} + L_{2} $$

4 $$ \left\{ {\begin{array}{*{20}c} {M_{1} = \frac{{w_{1} }}{2} + \frac{{w_{2} }}{2} - L_{1} } \\ {M_{2} = \frac{{w_{2} }}{2} + \frac{{w_{3} }}{2} - L_{2} } \\ \end{array} } \right. $$

### Three-stage analysis method

The construction of small clearances tunnel will generate repeated disturbance to the ground, and then further generate additional settlement on top of the surface settlement induced by the prior tunnel construction. However, this additional settlement is not simply the result of accumulating Peck's formula. In this paper, based on the Peck formula and the theory of repeated disturbance area calculation, the calculation method of surface settlement induced by three-hole tunnel excavation is proposed through a three-stage analysis. The calculation method needs to satisfy the following assumptions.

1. Assume that the surrounding soil is isotropic and homogeneous, and that the strata in which the three tunnels are located are uniformly distributed in the horizontal direction.
2. Assume that additional settlement from repeated disturbance of the two adjacent tunnels is distributed only on the ground surface between the two tunnels, and that no additional settlement occurs for the ground surface on the outside of the two tunnels.
3. The effect of grouting on surface deformation is not considered.

**Figure 3:**
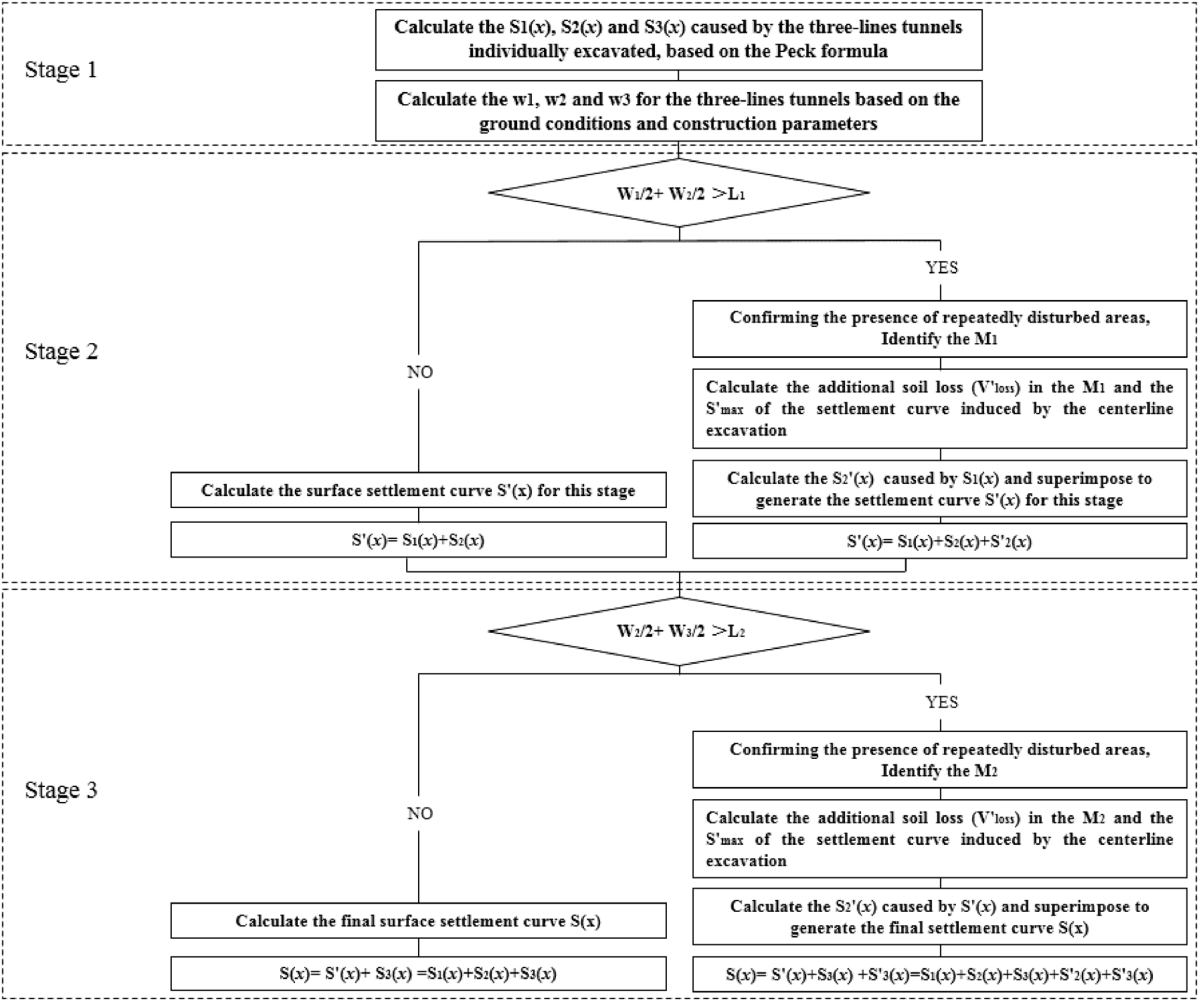
Flow chart of the "three-stage analysis" method.

The specific steps for calculating the surface settlement of a three-hole parallel tunnel using the "three-stage analysis" are shown in Fig. 3.

- *Stage 1* Calculation of standard surface settlement curves S_1_(x), S_2_(x) and S_3_(x) for three tunnels induced by separate excavations without considering the effect of repeated disturbances, respectively.
- *Stage 2* Determine whether the median tunnel will cause repeated disturbances to the soil. If there is no repeated disturbance then the surface settlement induced by the construction of two adjacent tunnels is directly superimposed on the space to form the final surface settlement curve. If there is disturbance, the soil loss ${V\mathrm{^{\prime}}}_{\text{1loss}}$ and the maximum settlement value ${S\mathrm{^{\prime}}}_{\text{1max}}$ in the repeatedly disturbed area need to be calculated from the disturbance range M_1_, and then the additional surface settlement S'_2_(x) induced by the repeated disturbance caused by the median excavation is calculated.
- *Stage 3* For the three-hole parallel tunnel, after obtaining the additional settlement generated by the excavation of the second intermediate line, the additional settlement S'_3_(x) generated by the settlement curve of this stage on the standard curve of surface settlement induced by the construction of the third tunnel is further calculated. Finally, the standard curve of surface settlement induced by the construction of the three tunnels is superimposed on the additional settlement of the surface induced by the construction of the intermediate line and the third tunnel, and the final surface settlement induced by the construction of the three tunnels is calculated.

If M < 0, it means that the spacing between two adjacent tunnels is large and additional settlement can be ignored, and the final surface settlement curve of surface settlement at this time only needs to be superimposed on the standard curve of surface settlement induced by the three tunnels individually excavated, as shown in Eqs. (5) and (6).

5 $$ {\text{S}}\left( {\text{x}} \right) = {\text{S}}_{\max 1} \cdot \exp \left[ {\frac{{ - ({\text{x}} - {\text{x}}_{1} )^{2} }}{{2{\text{i}}_{1}^{2} }}} \right] + {\text{S}}_{\max 2} \cdot \exp \left[ {\frac{{ - {\text{x}}^{2} }}{{2{\text{i}}_{2}^{2} }}} \right] + {\text{S}}_{\max 3} \cdot \exp \left[ {\frac{{ - ({\text{x}} + {\text{x}}_{2} )^{2} }}{{2{\text{i}}_{3}^{2} }}} \right] $$

6 $$ {\text{S}}_{{\max \cdot {\text{n}}}} = \frac{{\sqrt {\uppi } {\text{D}}^{2} {\text{V}}_{{{\text{ln}}}} }}{{4\sqrt 2 \cdot {\text{i}}_{{\text{n}}} }},\quad {\text{n}} = 1,2,3 $$

where ${\mathrm{S}}_{\mathrm{max}\cdot \mathrm{n}}$ is the maximum surface settlement value (mm) induced by the excavation of the nth tunnel alone; ${\mathrm{i}}_{\mathrm{n}}$ is the surface settlement trough width coefficient (m) induced by the excavation of the nth tunnel; ${\mathrm{V}}_{\mathrm{ln}}$ is the soil loss rate caused by the excavation of the nth tunnel; ${\mathrm{x}}_{1}$ is the lateral distance between the left tunnel and the center axis of the middle tunnel (m); x_2_ is the lateral distance between the right tunnel and the center axis of the middle tunnel (m); where ${\mathrm{V}}_{\mathrm{ln}}$ and ${\mathrm{i}}_{\mathrm{n}}$ can be obtained by inverse analysis using the measured prior tunnel surface lateral settlement data on site.

When M > 0, it indicates that there is a repetitive disturbance area between two adjacent tunnels, and additional settlement needs to be considered. Monitoring data from a large number of actual projects show that additional settlement from repeated disturbances can be fitted by Gaussian curves as well, so the characteristic curves of additional surface settlement can be expressed by Eqs. (7) and (8) as follows.

7 $$ {\text{S}}^{\prime } \left( {\text{x}} \right) = {\text{S}}^{\prime }_{{{\text{max}}}} \cdot \exp \left[ {\frac{{ - {\text{x}}^{{\prime {2}}} }}{{2{\text{i}}^{{\prime {2}}} }}} \right] $$

8 $$ {\text{S}}^{\prime }_{\max } = \frac{{{\text{V}}^{\prime }_{{{\text{loss}}}} }}{{{\text{i}}\sqrt {2\pi } }} $$

where S'(x) is the additional surface settlement (mm); ${\mathrm{S{\prime}}}_{\mathrm{max}}$ is the additional peak surface settlement (mm); x′ is the relative lateral distance between the ground calculation point and the tunnel axis (m); ${\mathrm{V{\prime}}}_{\mathrm{loss}}$ is the additional soil loss per unit length (m3/m); i′ is the additional settlement trough width coefficient (m).

Stallebrass et al.^24^ fitted a large amount of measured data and found a linear relationship between the additional settling trough width coefficient i' and the width of the repetitive disturbance zone M.

9 $$ i^{\prime} = M/5 $$

Dong et al.^25^ obtained the relationship between the prior tunnel soil loss and the additional unit length soil loss in the repeatedly disturbed area using least squares fitting.

10 $$ V^{\prime}_{loss} = 0.0249 + 0.2845V_{loss} $$

The additional settlement curves S'_2_(x) and S'_3_(x) can be obtained by substituting the results of Eqs. (9) and (10) into Eqs. (7) and (8), and finally the additional settlement can be superimposed on the standard surface settlement curve to obtain the final surface settlement induced by the construction of the three tunnels. Zeng^26^ once proposed a three-stage analysis method to predict the surface settlement of the tunnel, but it is not accurate enough to describe the calculation formula of the lateral disturbance range in the repeated disturbance area. Therefore, this paper improved the method and put forward an improved three-stage analysis method.

11 $$ \begin{gathered}   \text{S}\left( \text{x} \right) = \text{S}_{1} \left( x \right) + \text{S}_{2} \left( x \right) + \text{S}_{3} \left( x \right) + \text{S}^{\prime}_{2} \left( x \right) + \text{S}^{\prime}_{3} \left( \text{x} \right) = S_{{\max 1}}  \cdot \exp \left[ {\frac{{ - (\text{x} - \text{x}_{1} )^{2} }}{{2\text{i}_{1} ^{2} }}} \right] + \text{S}_{{\max 2}}  \cdot \exp \left[ {\frac{{ - \text{x}^{2} }}{{2i_{2}^{2} }}} \right] \hfill \\   \quad \quad \quad  + \text{S}_{{\max 3}}  \cdot \exp \left[ {\frac{{ - (\text{x} + \text{x}_{2} )^{2} }}{{2i_{3}^{2} }}} \right] + \text{S}^{\prime}_{{\max 2}}  \cdot \exp \left[ {\frac{{ - (\text{x}^{\prime2})}}{{2i^{\prime2}}}} \right] + \text{S}^{\prime}_{{\max 3}}  \cdot \exp \left[ {\frac{{ - (\text{x}^{\prime} + \text{x}_{2} )^{2} }}{{2\text{i}^{\prime2}}}} \right] \hfill \\  \end{gathered}  $$

## Overview of the study area

### Study interval overview

This paper relies on the project for a district tunnel of Nantong rail transit in Jiangsu, China. The interval tunnel is a treble-hole parallel tunnel, divided into upstream line, downstream line and parking line. The spacing between the upper and lower lines is 22m, the maximum slope of the line is 4%, the minimum slope is 2%, and the burial depth of the line is 8.916 ~ 9.946 m. The tunnel is excavated with Liaoning San San earth pressure balance shield machine. The excavation diameter is 6480 mm, using panel box type spokes type cutter, segment piece length 1200mm, outer diameter 6200 mm, inner diameter 5500 mm.

**Figure 4:**
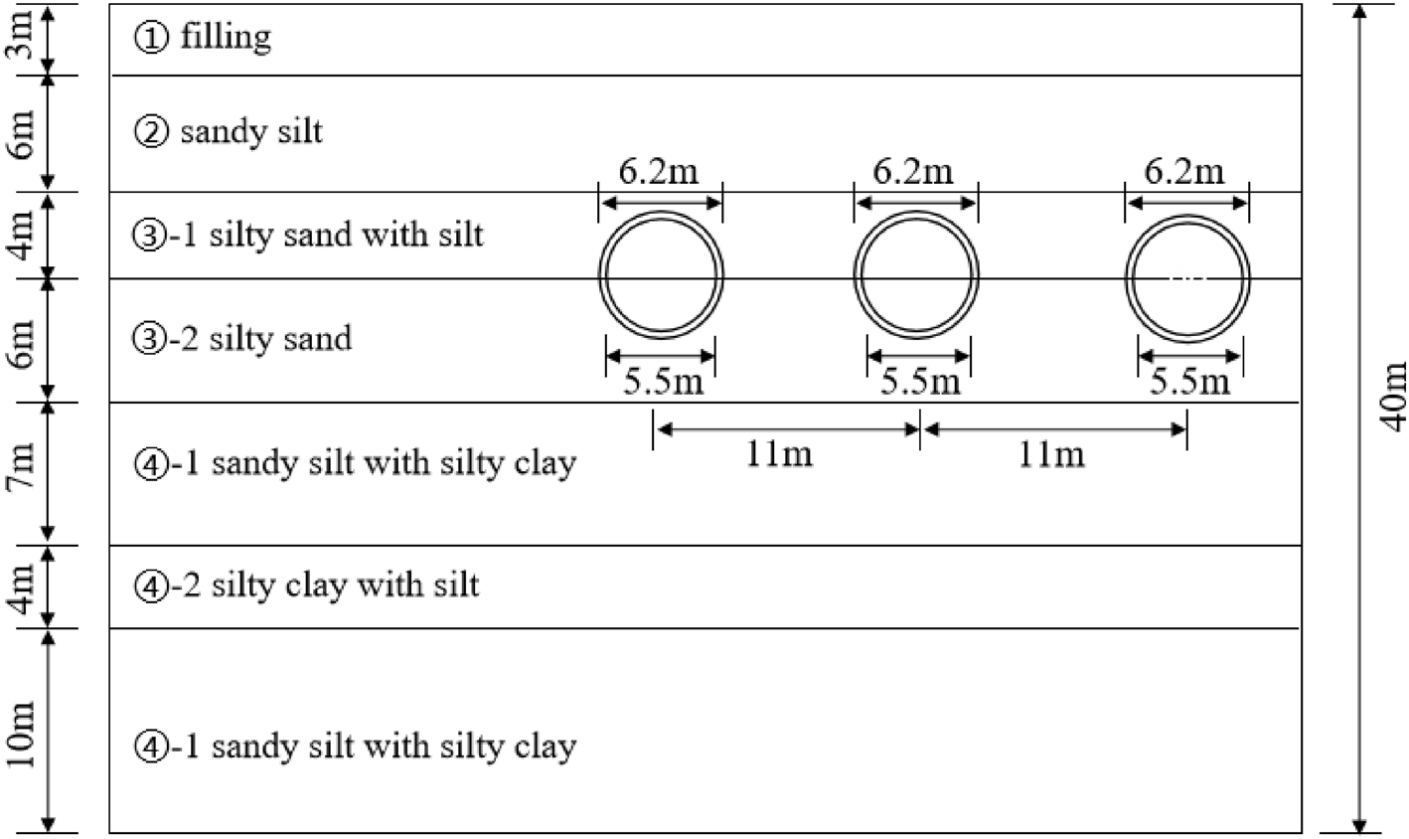
Geological profile.

The soft and weak soil layers along the line are thick, mainly powdered clay, chalk, fine sand, fine sand and medium-coarse sand. Figure 4 shows the stratigraphy of the study area divided into 7 layers from top to bottom. Among them, the stratum of the tunnel body in the interval is mainly ③-1 silty sand with silt and ③-2 silty sand.

The soil layer from top to bottom is ① filling (3 m), ② sandy silt (6 m), ③-1 silty sand with silt (4 m), ③-2 silty sand (6 m), ④-1 sandy silt with silty clay (7 m), ④-2 silty clay with silt (4 m), sandy silt sandy clay (10 m). The three tunnels are located in the depth of 13 m, spanning the silt sand sandy clay and silt layer, with the total thickness of soil layer of 40 m.

### Area monitoring point setting

**Figure 5:**
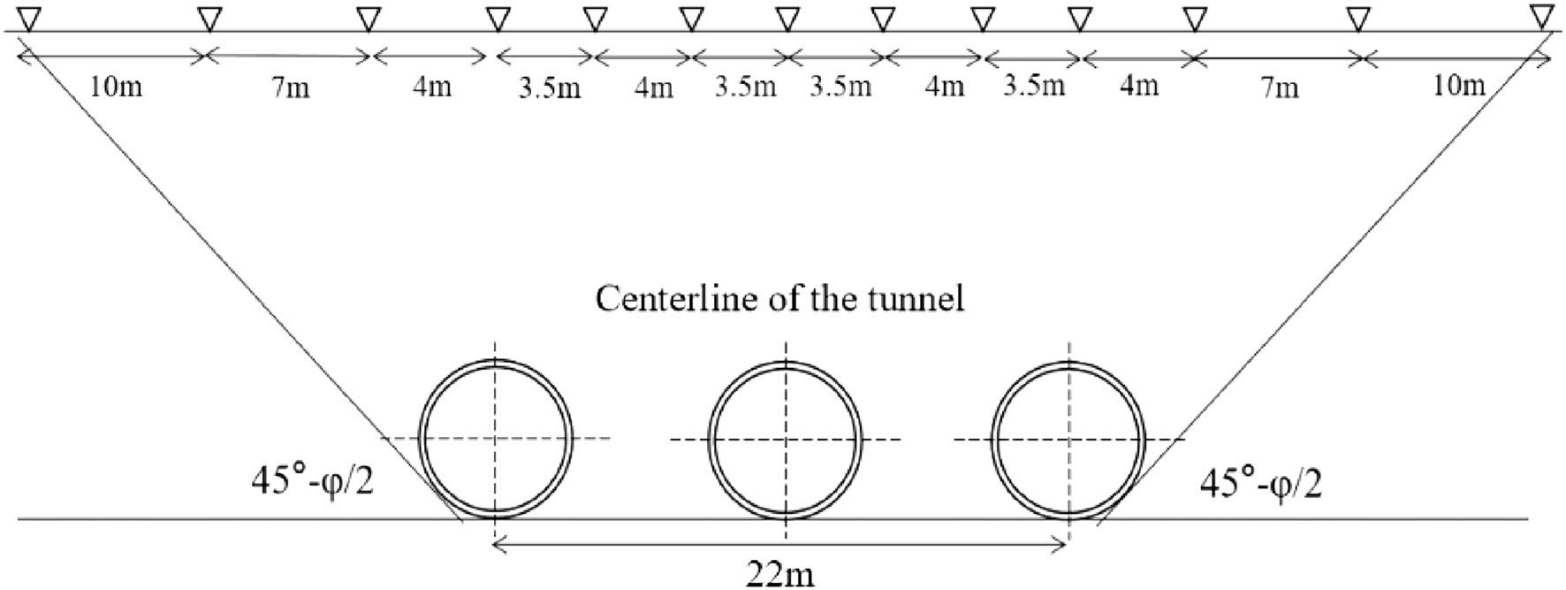
Monitoring point arrangement.

The interval is 516m in total, 1.2m per ring, 430 rings in total. For the 0th ~ 50th ring and the 380th ~ 430th ring, a monitoring section is set every 6m. For the 50th ~ 100th ring and the 330th ~ 380th ring, a monitoring section is set up every 12m. From the 100th ~ 330th ring, a monitoring section is set up every 24 m ~ 36 m. A total of 39 monitoring sections are set up in the whole area. Figure 5 shows the layout of each monitoring section.

## Numerical simulation and excavation sequence design

### Calculation model

The model was built based on Flac3d 6.0 finite difference software. The influence range of the tunnel excavation process is about 3 ~ 5 times the tunnel diameter, so the length and height of the model are determined as 100 m and 40 m respectively to mitigate the influence of boundary effects. According to the Nantong shield tunnel excavation, the shield tunnel segment piece is 1.2 m per ring. The width of the model is taken as the distance of 40 rings, which is 48 m.

Flac3d finite difference software was used for numerical simulation of the construction process of the three-hole tunnel. Mohr–Coulomb constitutive model was used to simulate in-situ soil mass, the contact model was Coulomb shear model, the excavation simulation method was zero model to simulate tunnel excavation, and the elastic model to simulate lining segments. The mesh was divided into tetrahedral mesh.

**Figure 6:**
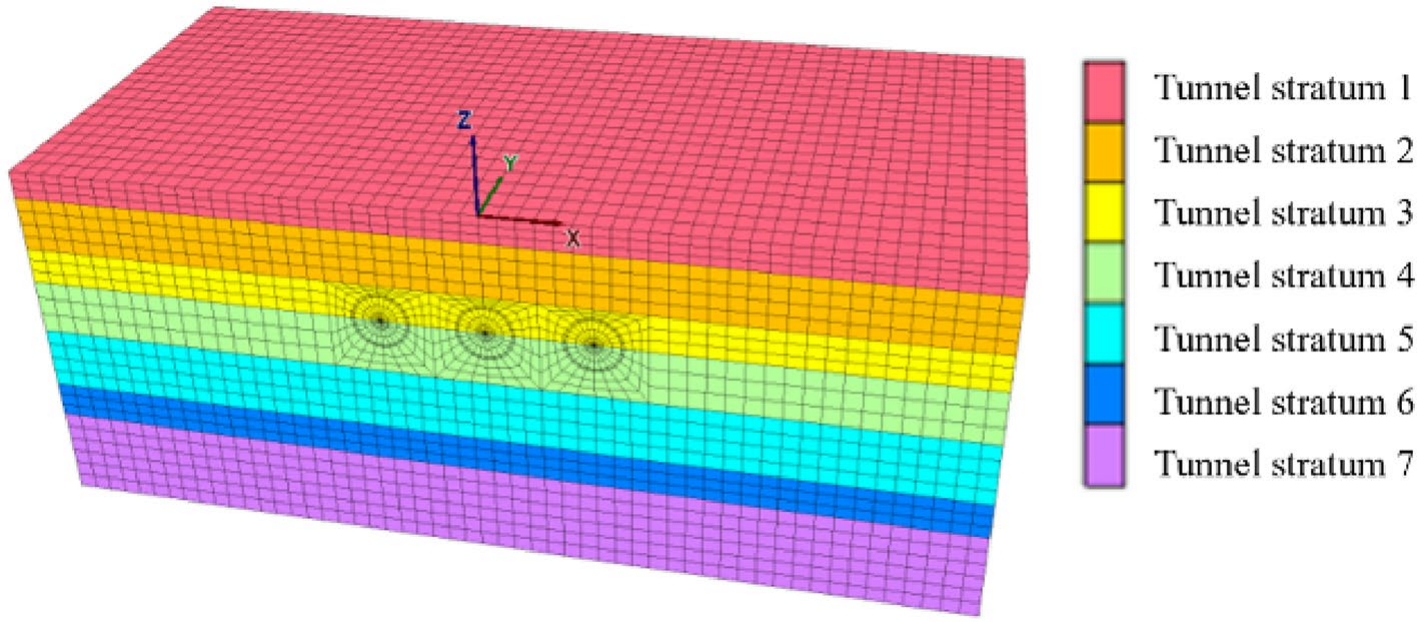
Stratigraphic group.

**Figure 7:**
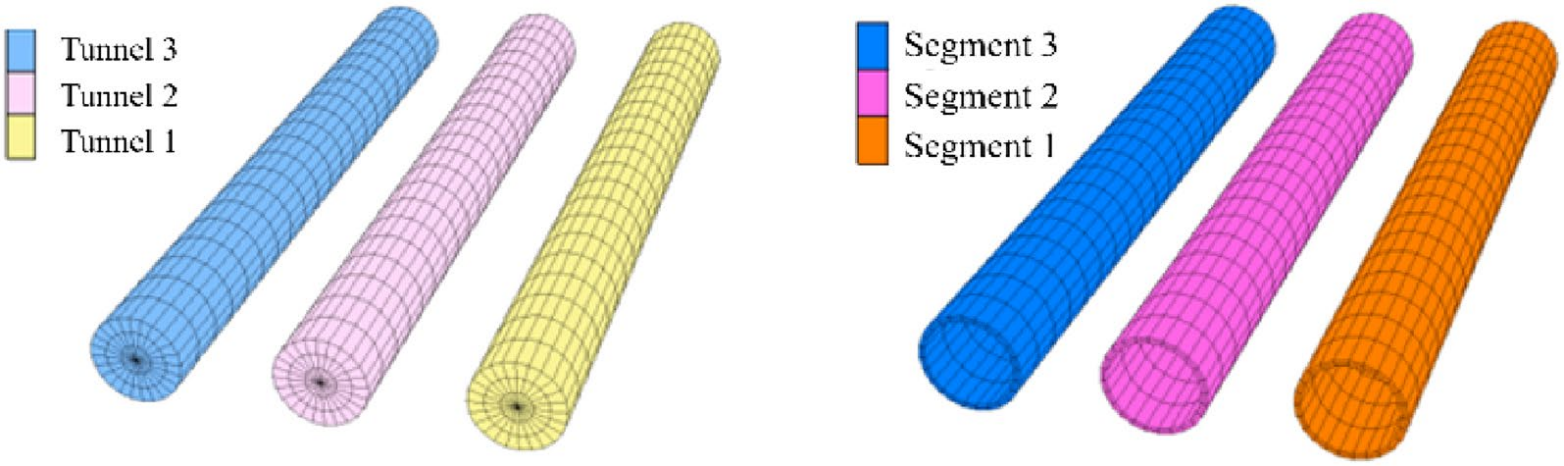
Tunnel and segment model grouping situation.

Figure 6 shows the numerical model grid division stratigraphic grouping. Figure 7 shows the grouping of tunnel and segment models. According to the actual situation, the layers are divided from top to bottom into Section 1–Section 7. According to the actual excavation sequence of Nantong, the tunnel grouping is Tunnel 1, Tunnel 2 and Tunnel 3 from right to left, and the corresponding liner segment grouping is Segment 1, Segment 2 and Segment 3 respectively. When the optimization scheme of tunnel excavation sequence is designed, the mesh sizes of the numerical models of different optimization schemes are consistent.

The top surface of the model is set as a free boundary. The remaining four faces are set with normal constraints in the x and y directions. The nodes at the bottom of the model are set as normal and tangential constraints. The load action is mainly considered as the self-weight load of the model.

#### Model parameters

According to the Nantong field monitoring data, the physical and mechanical parameters of each soil layer in the numerical simulation are shown in Table 1. The segment liner is 0.7 m, and C50 concrete material parameters are used, with a modulus of elasticity of 3.5 × 10^4 MPa, Poisson's ratio of 0.2 and mass density of 25KN/m^2^.

**Table 1:** Physical and mechanical parameters.

Layer	Thickness (m)	Weight (kN/m3)	Fric (°)	Cohesion (KPa)	Bulk (MPa)	Shear (MPa)
①	3.0	18.0	20.0	14.0	16.48	48.96
②	6.0	18.2	23.0	15.4	25.28	54.88
③-1	4.0	18.5	27.8	9.4	36.16	78.40
③-2	6.0	18.6	28.98	9.0	27.76	87.20
④-2t	7.0/10.0	18.0	18.4	16.2	18.88	40.96
④-2	4.0	18.2	19.2	13.0	32.20	57.60

### Monitoring point setting

**Figure 8:**
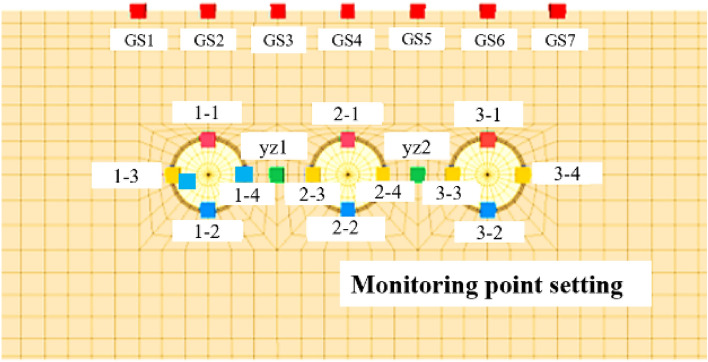
Model monitoring section monitoring point arrangement.

The numerical simulation mainly investigates the effects of three parallel shield tunnel excavations on surface settlement and Segment deformation under different excavation sequences. According to the actual placement of points in Nantong, points are placed in the numerical model for the top of the tunnel corresponding to the ground surface, the top of the segment, the bottom of the segment, and the two ends of the segment. There are 21 monitoring points in total, as shown in Fig. 8.

### Excavation sequence design

In the process of numerical simulation, each tunnel excavation was performed in one excavation step of 2 times the length of the segment. Every 2.4m excavation step, the model width is 48m and there are 20 rings. Since the last step requires additional liner, 21 excavation steps are required. In this paper, we only consider the case of tunnels in sequence, so we design four working conditions: "sequential tunnel excavation", "excavation of both sides of the tunnel first and then the center tunnel", "excavation of the center tunnel first and then both sides of the tunnel", and "S-type sequential excavation", as shown in Table 2**.**

**Table 2:** Design of treble-hole parallel tunnel excavation sequence.

Case	Excavation sequences	Schematic
Case 1	Right–center–left	
Case 2	Right–left–center	
Case 3	Center–right–left	
Case 4	Right–center (reverse)–left	

### Numerical simulation results

#### Surface settlement results

Based on the numerical simulation study of the surface settlement induced by the shield construction of three tunnels in the study area, the surface settlement distribution patterns caused by different construction stages were firstly analyzed qualitatively through the surface settlement cloud maps, so the surface settlement distribution cloud maps of different cavities under four excavation sequences of Case 1, Case 2, Case 3 and Case 4 were drawn in a total of six stages of excavation at 24m and penetration.

(1) The vertical settlement pattern of the ground surface in Case 1

**Figure 9:**
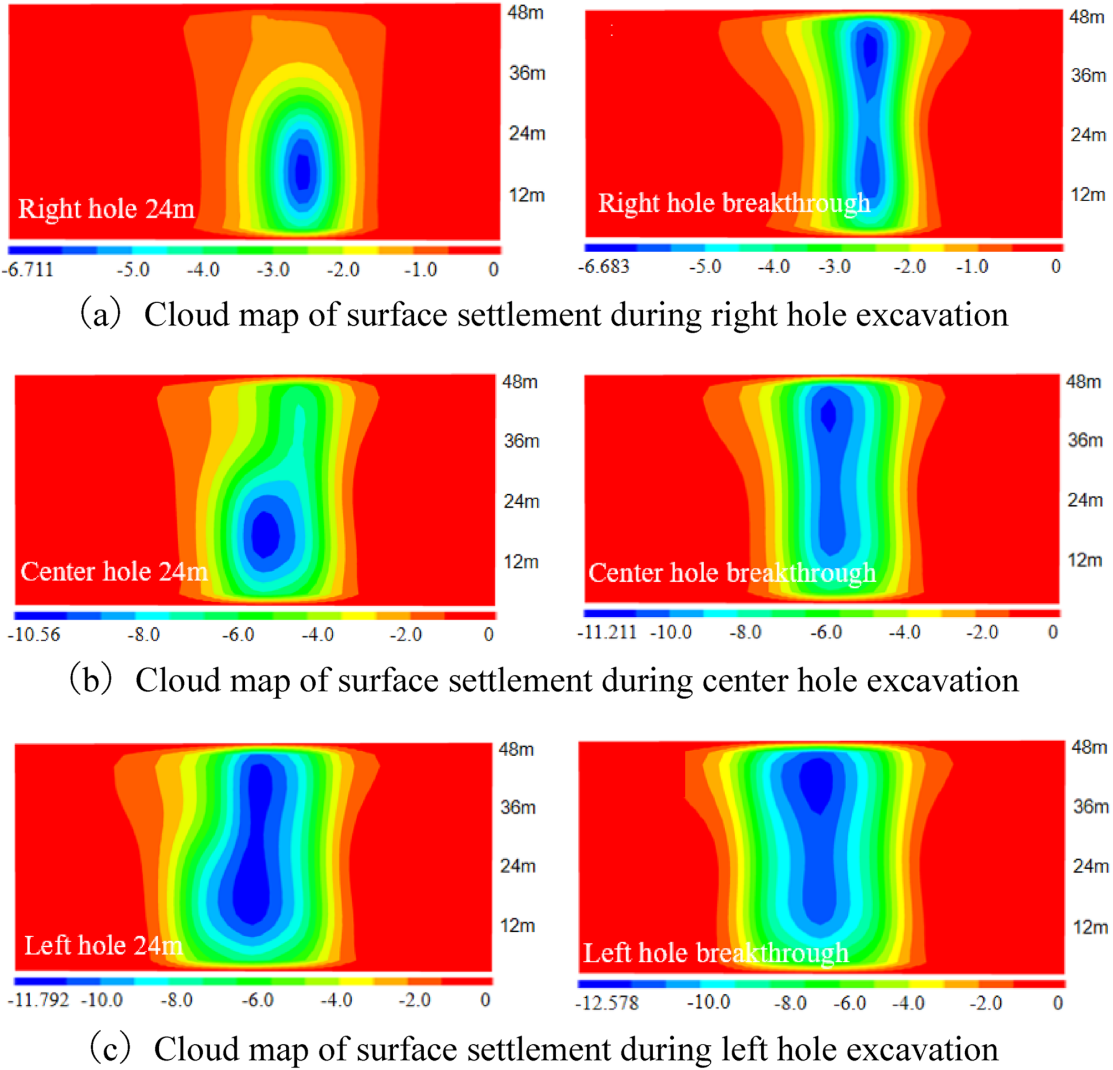
Ground settlement displacement clouds during excavation in Case 1 (mm).

The surface settlement clouds of the six stages of Case 1 are shown in Fig. 9, which can clearly reflect the change law of surface settlement caused by shield excavation in Case 1. As the shield machine advances, the surface settlement also advances, and the settlement caused by shield excavation is ahead of the location of the shield machine. In the center hole excavation process, the settlement distribution of the ground surface spreads significantly to the left. However, since the excavation of the right hole, which was constructed first, had already disturbed the original soil layer, the settlement of the ground directly above the tunnel axis during the center hole excavation was slightly larger than that of the ground directly above the tunnel axis of the right hole. During the excavation of the left cavern, the settlement distribution of the ground surface continued to spread to the left, and the maximum value of settlement was gradually shifted to the upper part of the center cavern after the excavation surface was passed.

**Figure 10:**
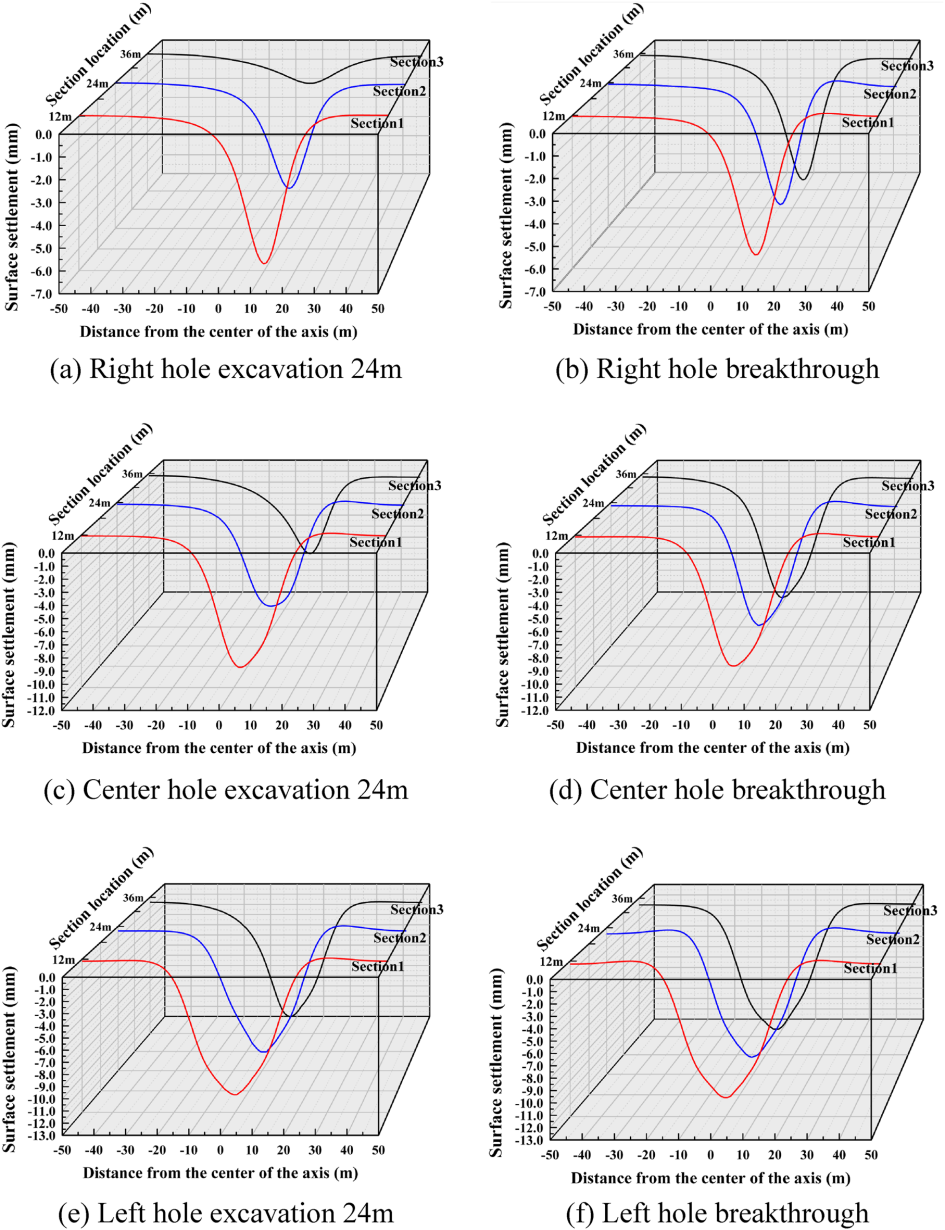
Settlement troughs in each monitoring section during the excavation process of Case 1.

It is drawn into a three-dimensional settlement trough change schematic diagram as shown in Fig. 10, and the monitoring sections selected in the figure are the three cross sections at y-coordinates of 12 m, 24 m and 36 m, respectively. For Case 1, i.e., the actual excavation situation, it can be seen from Fig. 10a and b that with the excavation of the right hole, surface settlement occurs sequentially in the three cross sections, and the distribution range of the settlement trough in the lateral direction is about 20m (3.2D) on each side. When the shield machine excavated to section 1, both section 2 and section 3 started to show trace settlement, indicating that tunnel excavation would also cause settlement difference in a certain range for the front end of its excavation direction. Therefore, in the actual excavation process, early warning should be done in advance.

With the excavation of the center hole, the maximum settlement value of the settlement trough began to gradually shift to the center, and the settlement trough showed a partial "V" shape. After the center hole was opened, the maximum settlement value of the tunnel appeared in section 3, with a peak value of 11.211mm, an increase of 69.49% compared to the peak settlement value of the right hole.

Figure 10e and f further plot the changes of the surface settlement trough caused by the start of excavation in the left hole. It can be seen that the overall change trend of the settlement trough curve is not significant. According to Fig. 10d, the cumulative surface settlement reached a peak of 11.8 mm at section 3 after the passage of the center hole, which is only a 7% increase compared to the peak settlement of the center hole.

(2) The vertical settlement pattern of the ground surface in Case 2

**Figure 11:**
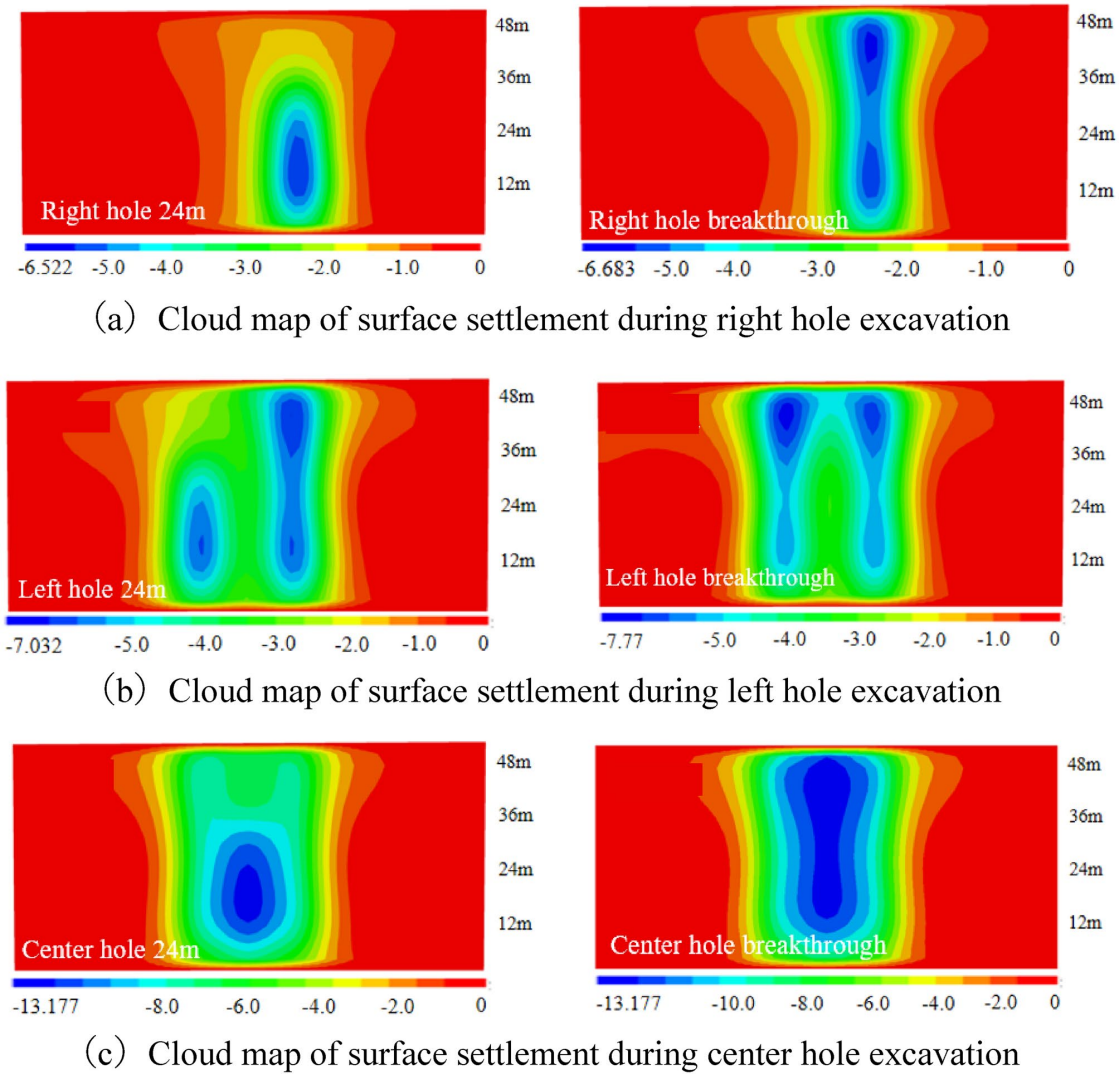
Ground settlement displacement clouds during excavation in Case 2 (mm).

The surface settlement clouds for the six stages of Case 2 are shown in Fig. 11. For Case 2, the settlement pattern of the right hole excavation remains the same as that of Case 1, and both indicate that the tunnel excavation will cause over-settlement of the soil. Since the net distance between the right and left tunnel is 15.8m, which is about 2.5D, the distance between the two holes is far apart. The settlement range in the left hole and the right hole overlap less, so there is no offset of maximum settlement. The maximum settlement of both left and right holes appeared directly above their tunnels, and the difference between them was not large, with the settlement of the later tunnel slightly higher than that of the earlier tunnel. During the center tunnel excavation process, the maximum settlement point shifted to directly above the center tunnel.

**Figure 12:**
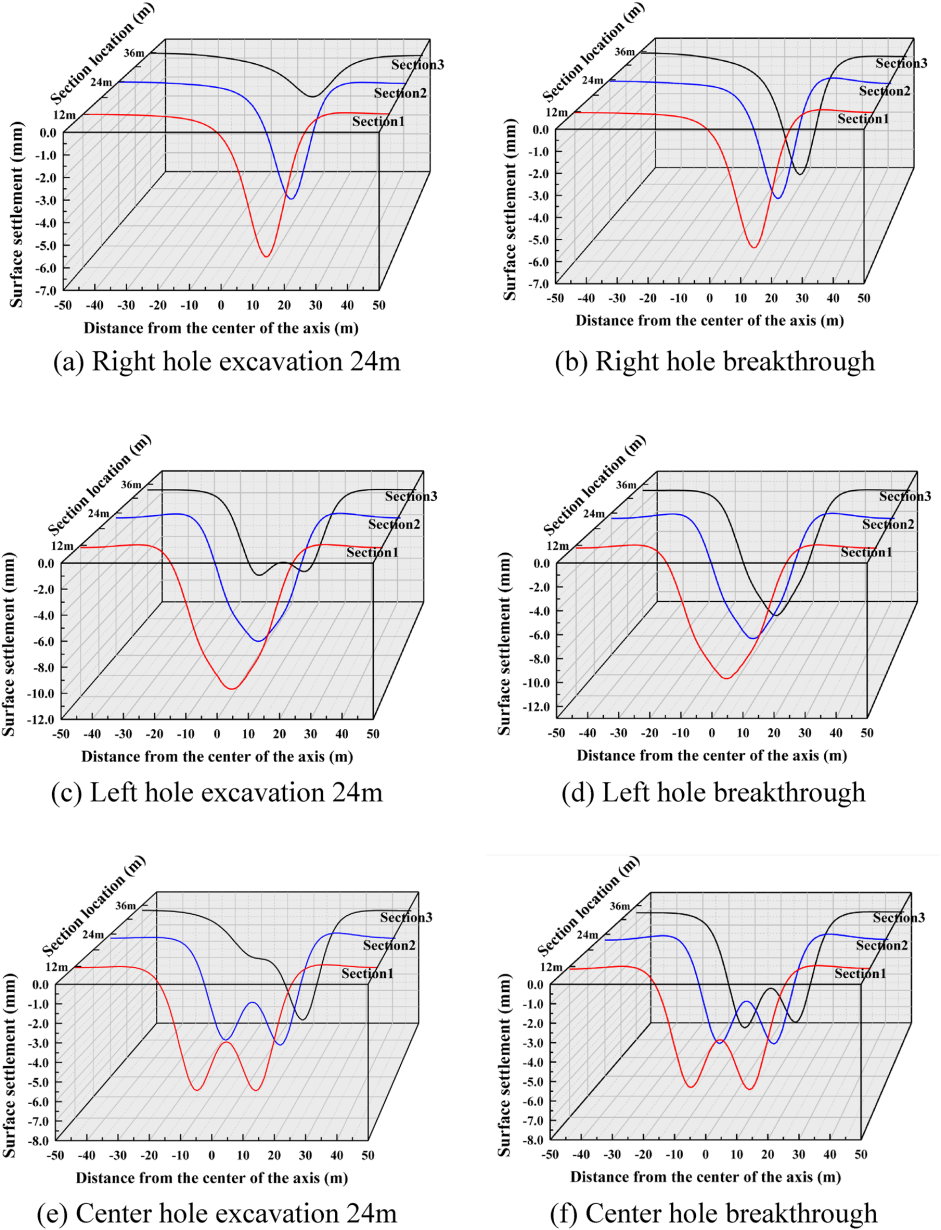
Settlement troughs in each monitoring section during the excavation process of Case 2.

A schematic diagram of the variation of the three-dimensional settlement trough is drawn as shown in Fig. 12. For Case 2, the excavation of the first tunnel is the same as that of Case 1. From Fig. 12c and d, it can be seen that the maximum settlement above the right tunnel of the first tunnel is 6.96 mm, and the maximum settlement above the left tunnel of the later tunnel is 7.29 mm. The surface settlement above the axis caused by the left tunnel excavation is slightly larger than that caused by the first right excavation with similar values, and the settlement trough caused by the superposition of the two exhibits a "W " shape, which is consistent with the results of most existing studies. Figure 12e and f represent the settlement pattern of the surface caused by the excavation of the third tunnel. After the excavation of the left and right caverns was completed, the excavation of the center cavern further excavated its intermediate rock columns, which would certainly cause more disturbance. The equilibrium that was restored after the completion of slurry lining in the left and right holes was broken again, and the soil structure that was reconsolidated in the first period was further destroyed, so the accumulated surface settlement caused by the center hole construction was further aggravated. Finally, it showed a big "V" shape. After the completion of the center tunnel, the surface settlement value reached a peak of 12.69mm in section 3, which was 74% higher than the peak settlement after the excavation of the second tunnel.

(3) The vertical settlement pattern of the ground surface in Case 3

**Figure 13:**
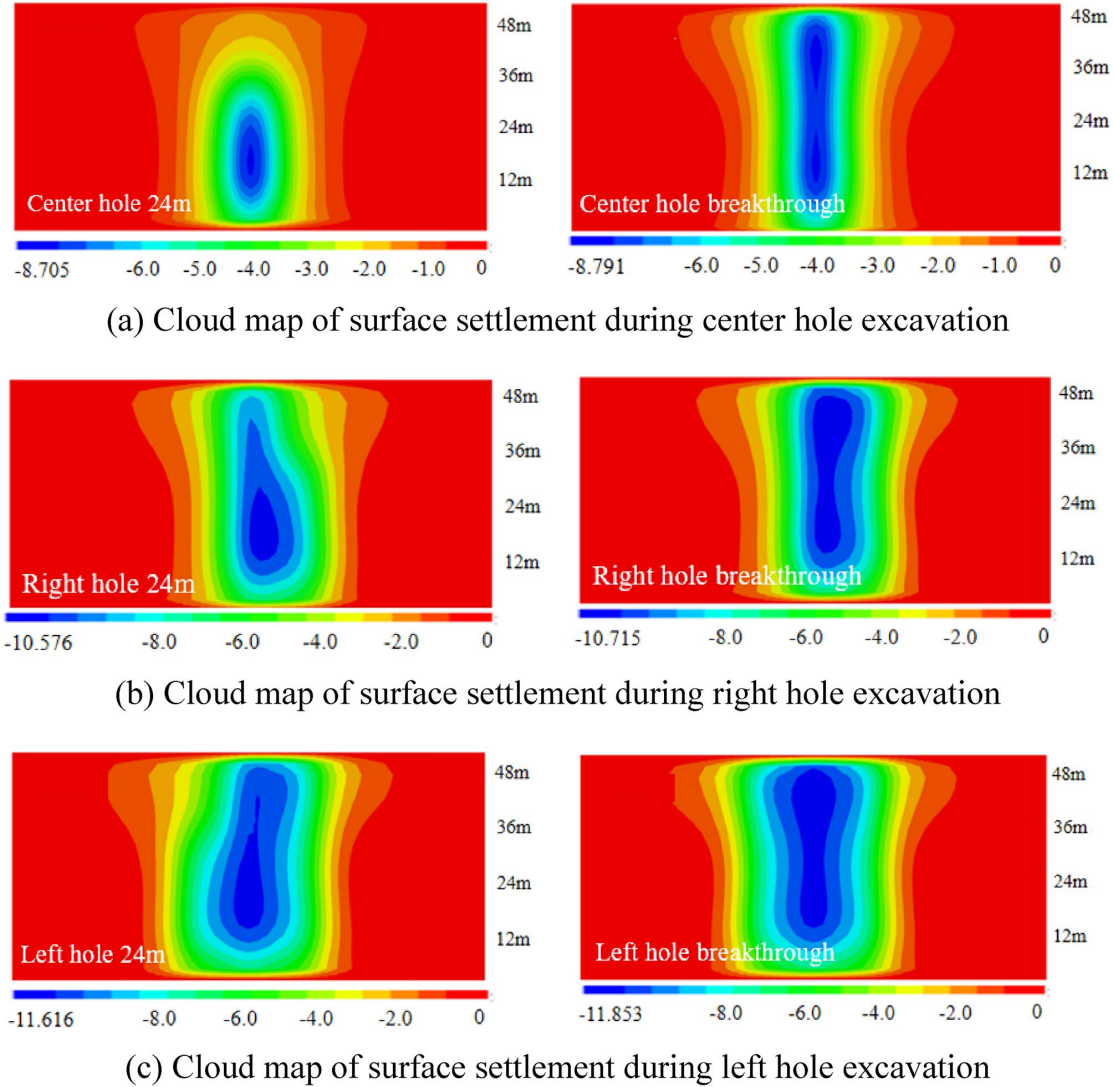
Ground settlement displacement clouds during excavation in Case 3 (mm).

The surface settlement clouds for the six stages of Case 3 are shown in Fig. 13. The maximum settlement value of the ground surface after the center cavern is 8.791mm, which is slightly larger than the maximum settlement value of the single cavern in the preceding tunnels of Case 1 and Case 2. When the right hole is excavated, the influence of surface settlement spreads to the right and the position of the maximum settlement point is shifted in the direction of the back tunnel. When the left hole is excavated, the influence range of surface settlement spreads to the left, and the position of the maximum settlement point gradually returns to the upper part of the center hole.

**Figure 14:**
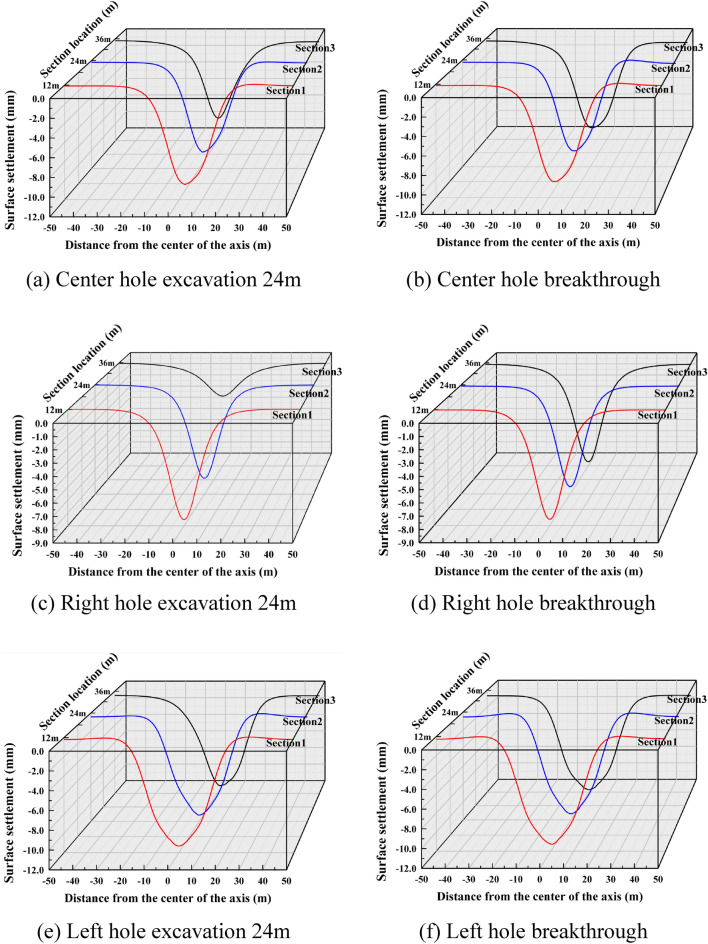
Settlement troughs in each monitoring section during the excavation process of Case 3.

The three-dimensional settlement trough is plotted as shown in Fig. 14. From Fig. 14c and d, it can be seen that the settlement trough curve caused by the excavation of the second tunnel, i.e., the right hole, is shifted to the right, showing a partial "V" shape. The maximum settlement value is 10.62mm after the right tunnel is opened, which is 26.58% higher than the peak settlement caused by the single tunnel excavation. Figure 14e and f further plot the settlement trough variation curve caused by the excavation of the third tunnel, the left tunnel. After the excavation of the left tunnel, the width of the settlement trough expands to the left, and the shape of the settlement trough gradually changes from partial "V" to deep "V", with the overall symmetry of left and right centered at x = 0. The final maximum settlement value obtained after all three holes were penetrated was 11.73 mm, which was 10.45% higher than the peak settlement value of the two holes penetrated.

(4) The vertical settlement pattern of the ground surface in Case 4.

**Figure 15:**
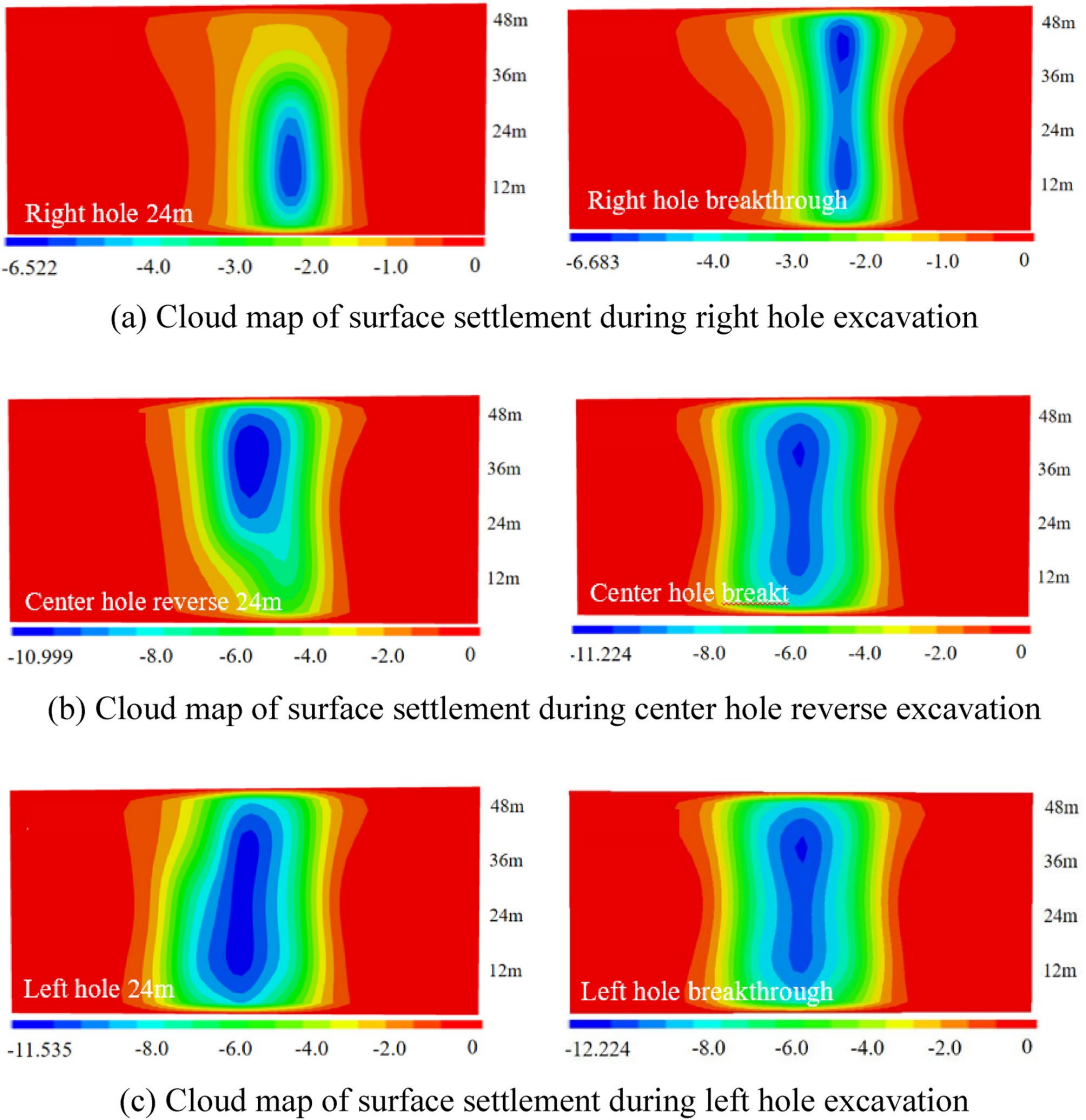
Ground settlement displacement clouds during excavation in Case 4 (mm).

The surface settlement clouds for the six stages of Case 4 are shown in Fig. 15. Since the only difference between the tunneling sequence and Case 1 is that the shield machine reverses the excavation direction, and all other conditions are the same, the diffusion cloud pattern is the same as that of Case 1. The diffusion cloud pattern is the same as that of Case 1. The diffusion is from the upper part of the right hole to the left.

**Figure 16:**
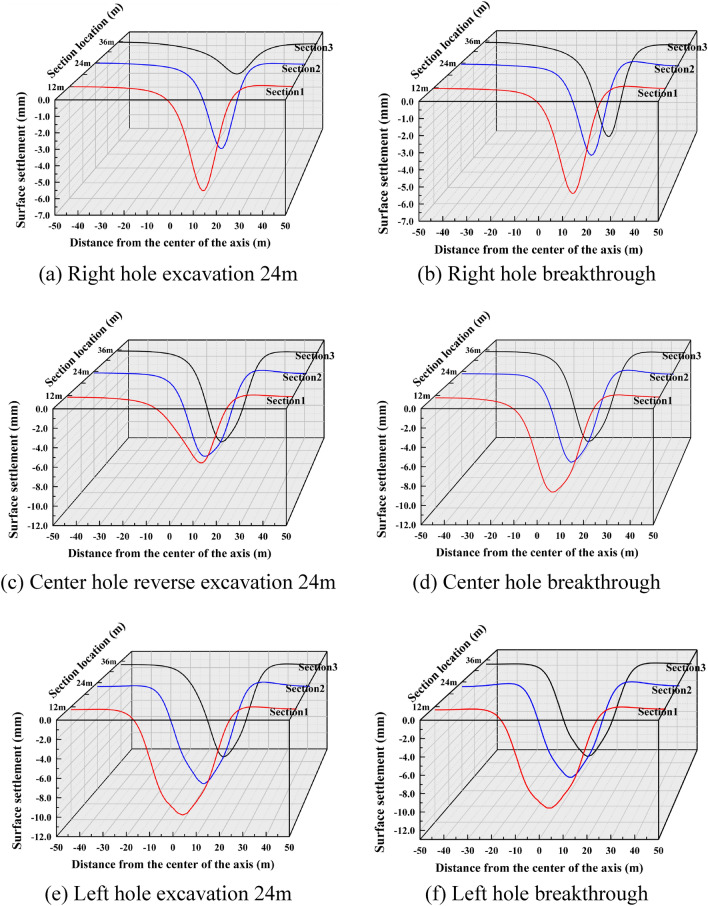
Settlement troughs in each monitoring section during the excavation process of Case 4.

The three-dimensional settlement trough is plotted as shown in Fig. 16. From Fig. 16c and d, it can be seen that the settlement trough curve caused by the second tunnel excavation also shows a partial "V" shape, with a maximum settlement value of 10.25 mm. Compared with the peak settlement increase of 73.14% caused by the single hole excavation. From Fig. 16e and f, it can be seen that after all the three holes are completed, the settlement trough is in the same large "V" shape as the first three working conditions, and the maximum settlement value is 11.41 mm. Compared with the peak settlement increase of 11.32% for the double holes.

(5) longitudinal ground surface settlement profile of analysis results

**Figure 17:**
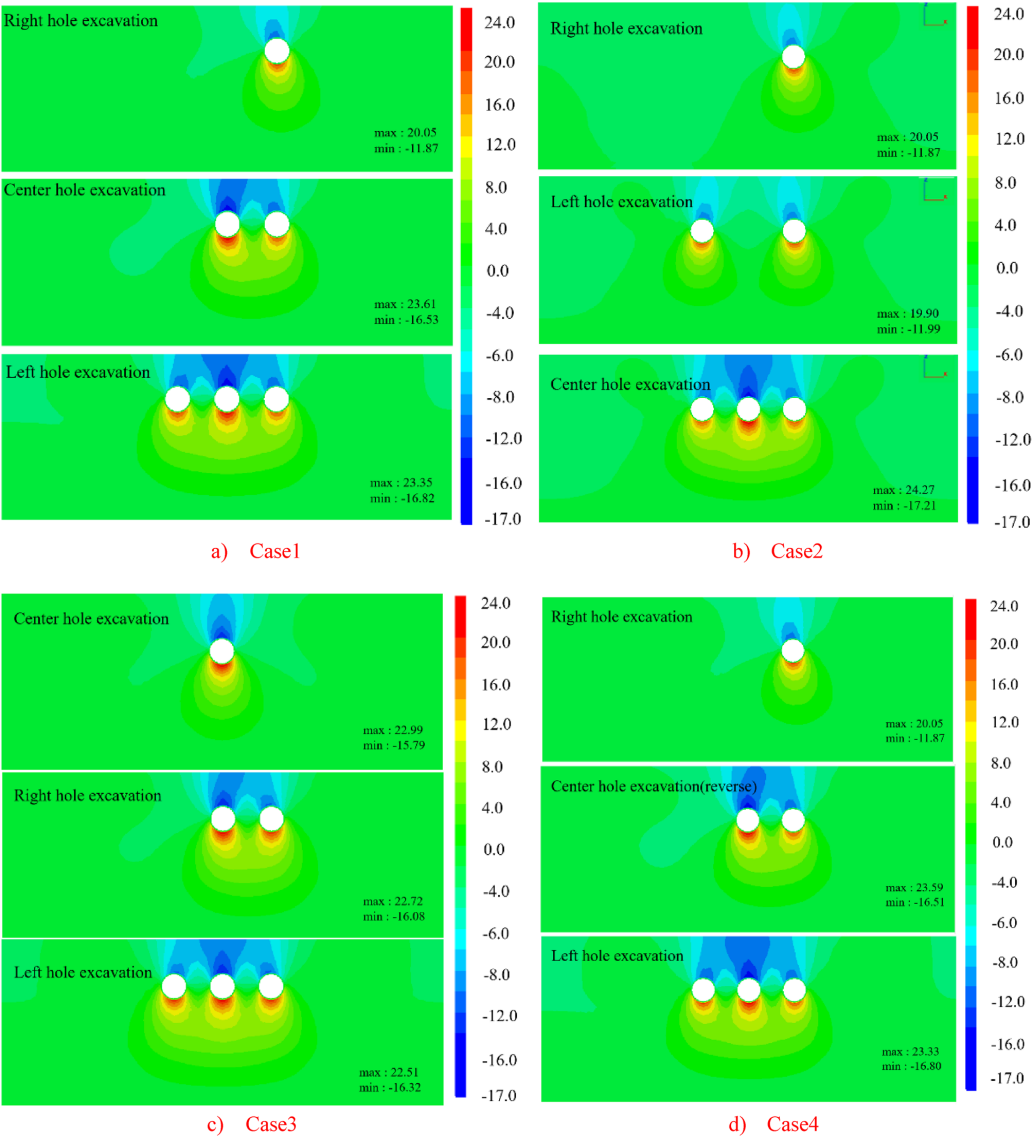
Longitudinal displacement cloud map of the surface.

The vertical displacement cloud image of each tunnel after penetration under four Case is shown in the Fig. 17. The maximum and minimum values under each state are extracted. It can be seen from the figure that the maximum vertical displacement of soil around the tunnel occurs at the position of the vault and the bottom of the tunnel, with downward vertical settlement at the vault and upward arch at the bottom. The maximum vertical displacement occurs at the arch top and arch bottom. For Case 1, the maximum settlement at the arch top is − 16.82 mm and the maximum uplift at the bottom of the arch is 23.35 mm. For Case 2, the maximum settlement at the arch top is − 17.21 mm, and the maximum uplift at the arch bottom is 24.27 mm. For Case 3, the maximum settlement at the arch top is − 16.32 mm, and the maximum uplift at the arch bottom is 22.51 mm. For Case 4, the maximum settlement at the vault is − 16.80 mm and the maximum uplift at the bottom of the arch is 23.33 mm. To sum up, the surrounding soil settlement and uplift are ranked from smallest to largest as follows: Case 3 < Case 4 < Case 1 < Case 2.

(6) Comparison of analysis results

**Figure 18:**
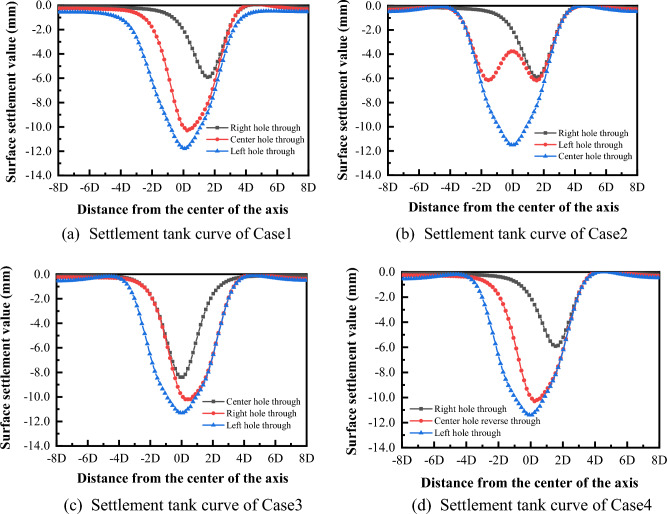
Variation law of settlement tank under four cases.

Comparison of the final surface settlement trough curve variation patterns for the four cases is shown in Fig. 18.

**Figure 19:**
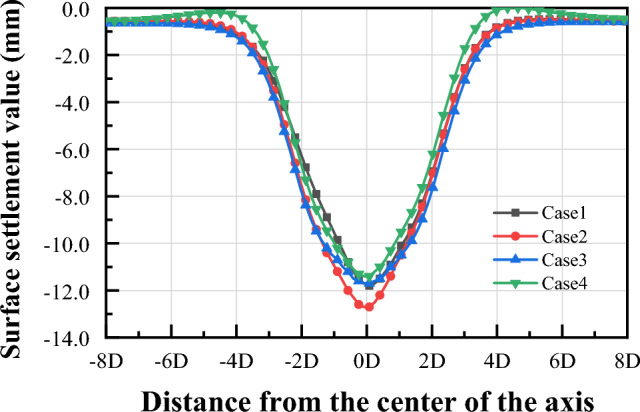
Comparison of the final shape of the settling tank for the four cases.

The final settlement trough curves for each case are shown in Fig. 19. From the figure, it can be seen that the final settlement troughs caused by different excavation sequences are similar in shape and are all large "V" shaped. The width of the settlement trough is basically the same, about 60 m (9D), but the maximum settlement value caused by the ground surface is different. The maximum surface settlement value for Case 2 is 12.69 mm, while the surface settlement values for Case 1 and Case 3 are similar, 11.8 mm and 11.73 mm respectively, and the minimum surface settlement value for Case 4 is 11.41 mm.

The reason for this difference is that the excavation sequence of "right-left-center" will cause more disturbance after the excavation of the right and left holes is completed, and the excavation of the center hole will cause further excavation of the center column, and the equilibrium that was restored after the completion of the grouting lining of the left and right holes will be broken again, and the soil structure that was reconsolidated in the first period will be further destroyed. The four options are ranked from smallest to largest in terms of settlement value: Case 4 < Case 1 ≈ Case 3 < Case 2.

#### Segment deformation results

In parallel tunnel excavation, the analysis needs to focus on the segment convergence of the preceding tunnel, so the horizontal and vertical displacement of the inner wall after the segment lining needs to be analyzed separately. For Case 1, Case 2 and Case 4, the analysis should focus on the impact on the right lining segment during the back tunneling process. For Case 3, the analysis should focus on the influence of the back tunneling process on the center lining segment. Different excavation sequences have different effects on the horizontal and vertical displacements of the first tunnel segment.

(1) Horizontal displacement of Segment

**Figure 20:**
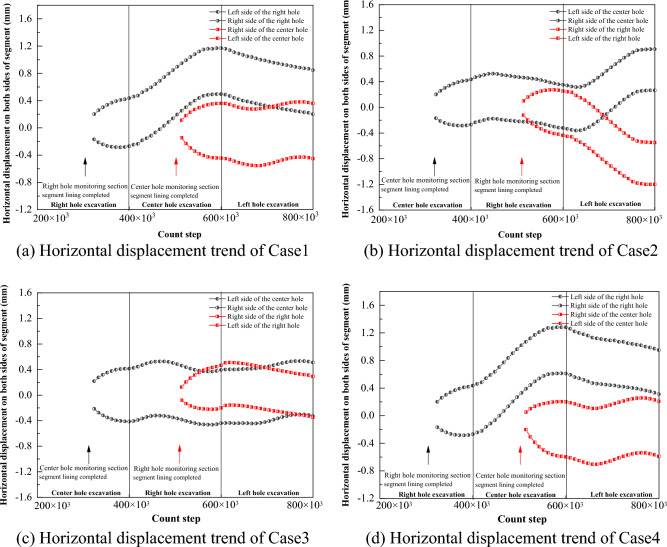
Trend of horizontal displacement during tunnel construction.

For the horizontal displacement of the tunnel segment, the common feature is that after the completion of the first tunnel segment lining until the first tunnel is completed, the left side of the tunnel moves to the left and the right side of the tunnel moves to the right, as shown in Fig. 20. When the second and third tunnels are excavated, the degree of convergence of the first tunnel segment remains stable and the tunnel as a whole is shifted to the left or to the right. For Case 1 and Case 4, the first tunnel is shifted to the right during the second tunneling process. In the third tunnel, the first tunnel is shifted to the left. For Case 2, the first tunnel is shifted to the left during the second tunneling process and to the right during the third tunneling process. For Case 3, the first tunnel moves to the right and then to the left during the second tunneling process, and the overall offset is not significant after the tunnel is completed, while the first tunnel moves to the left and then to the right during the third tunneling process.

(2) Vertical displacement of Segment

**Figure 21:**
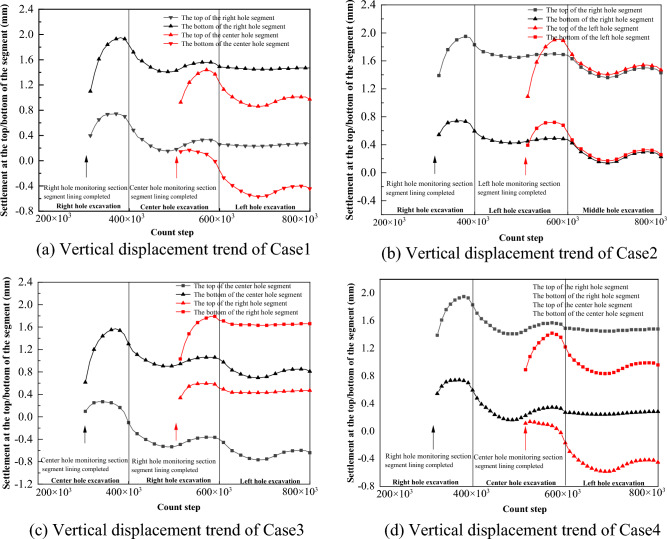
Trend of vertical displacement of segment during tunnel construction.

As can be seen from Fig. 21, before the first tunnel was excavated through the monitoring section until it was completed, the arch top and arch bottom of the right cavern produced arching, and the amplitude of arch bottom arching was larger than that of the arch top. Before the second and third tunnels were excavated to the monitoring section, the top and bottom of the arch of the right cavern both produced downward settlement. After the second and third tunnels were excavated to the monitoring section, the top and bottom of the arch of the right cavern produced a slight amount of arching again.

The difference of different excavation sequences on the settlement of the vault of the first tunnel is reflected in the magnitude of settlement and arching when excavating to the front and back part of the monitoring section. When the distance between the tunnel and the prior tunnel is farther, the change amplitude is smaller, and the change range is controlled within 0.2 mm. As in Case 1, Case 2 and Case 4, the change amplitude of the right tunnel segment when the left tunnel is excavated. On the contrary, the variation is larger, and the variation range is controlled within 0.2 ~ 0.4 mm, such as the variation range of the right segment in the center hole excavation in Case 1 and Case 4, and the variation range of the center hole segment in the left and right hole excavation in Case 3.

**Figure 22:**
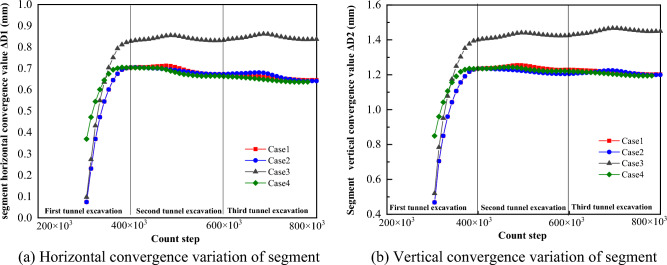
Trend of segment convergence during tunnel construction.

The horizontal convergence values of the segment can be calculated by subtracting the horizontal displacements of monitoring points 3 and 4 in the four cases, and the vertical convergence values of the segment can be calculated by subtracting the horizontal displacements of monitoring point 1 and 2. From Fig. 22, it is obvious that the vertical and horizontal convergence values of the segment of Case 1, Case 2 and Case 4 are similar, with the horizontal convergence in the range of 0.65 mm ~ 0.7 mm and the vertical convergence in the range of 1.2 mm ~ 1.3 mm. With the excavation of the back tunnel, the horizontal convergence of the segment slowly decreases, and the vertical convergence of the segment does not change much. The horizontal and vertical convergence values of the segment in Case 3 are significantly larger than the other three cases, with the horizontal convergence value in the range of 0.82 mm ~ 0.9 mm and the vertical convergence value in the range of 1.4 mm ~ 1.5 mm. The horizontal and vertical convergence values of the segment are slightly increased due to the excavation of the back tunnel. In summary, the horizontal and vertical convergence are ranked from smallest to largest: Case 1 ≈ Case 2 ≈ Case 4 < Case 3.

**Figure 23:**
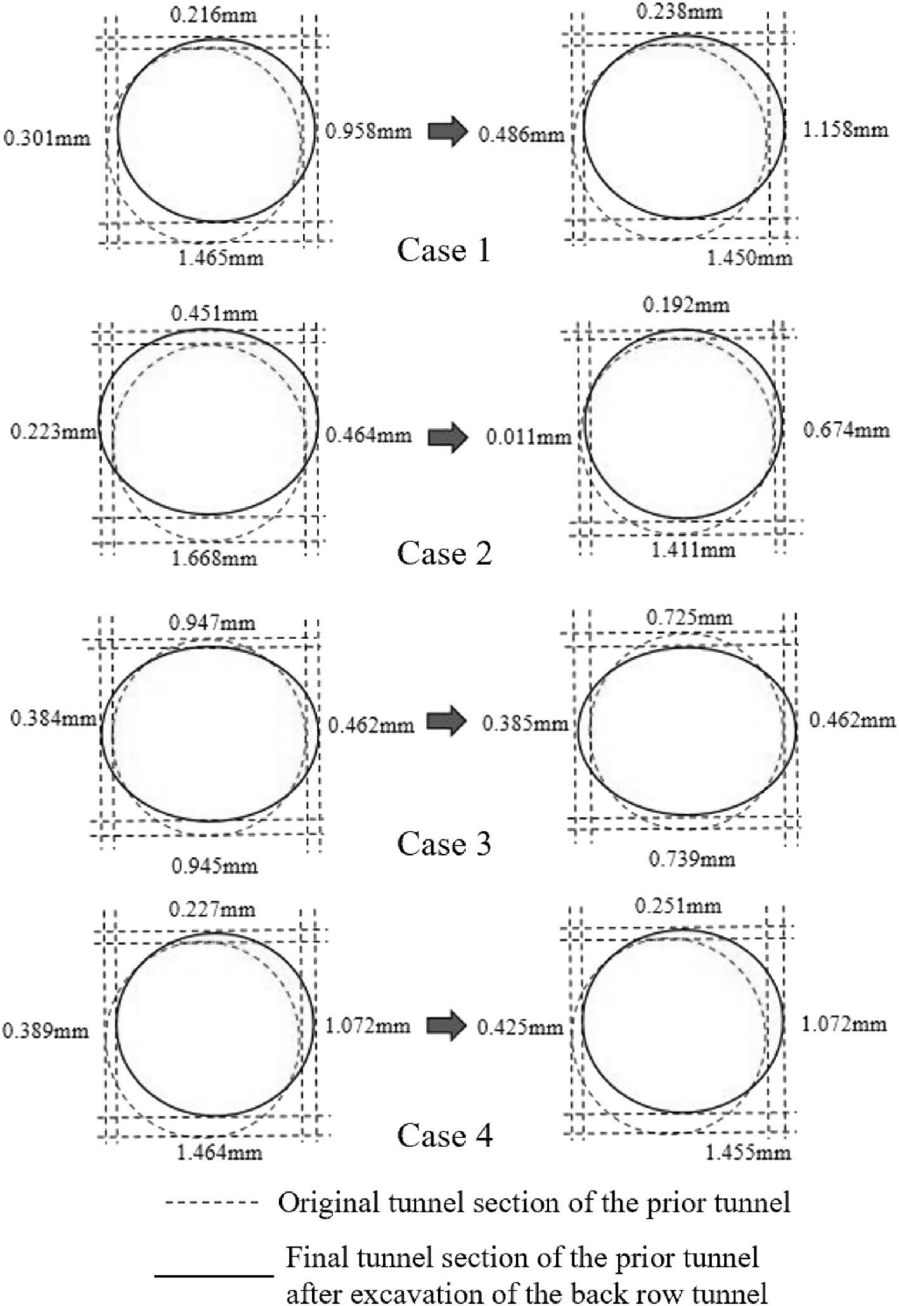
Schematic diagram of convergence deformation of the prior tunnel segment.

Comprehensive analysis of the vertical displacement of monitoring point 1 and 2 and horizontal displacement of monitoring points 3 and 4 of the prior cavern segment under the four cases can lead to the deformation diagram of the segment as shown in Fig. 23**.** The tunnel under all four cases changed from a standard round shape to an oval shape with a flat top and bottom and wide ends. With the subsequent tunnel excavation, the first tunnel in Case 1 and Case 4 continued to shift to the upper right, while the first tunnel in Case 2 shifted first to the upper and then to the right. Case 3 was not shifted in the center but was flattened more significantly than the other cases, so the convergence value was larger.

### comparative analysis results

**Figure 24:**
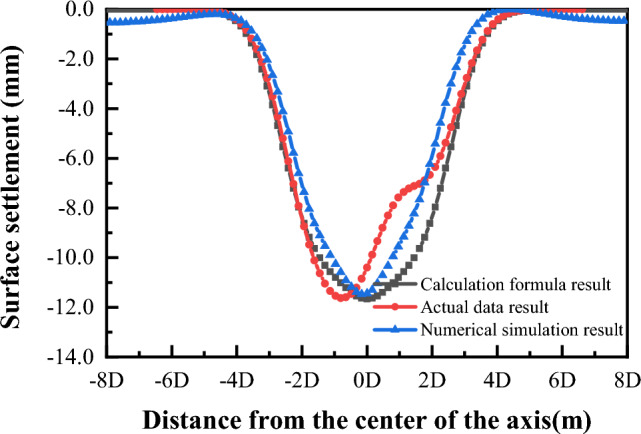
Comparison of surface settlement caused by tunnel excavation in three holes.

The actual construction plan of Nantong subway in Jiangsu Province, China, adopts the above-mentioned sequence of Case 1, i.e., "right-center-left" sequence. In the numerical simulation, Case 1 corresponds to the actual excavation sequence in Nantong, so the surface settlement slots of section 2 in each stage of Case 1 are extracted. The settlement curves calculated by the "three-stage analysis method" and the settlement curves fitted by the field measurements were compared, as shown in Fig. 24**.**

As can be seen from Fig. 24, the width of the settlement trough and the maximum settlement amount obtained by the three methods are basically the same. The maximum settlement value obtained from the numerical simulation is − 11.8 mm, and the maximum settlement value predicted by the "three-stage analysis method" is − 11.66 mm, with an error of − 11.63 mm when compared with the actual measured settlement value of − 11.63 mm. 1.46% and 0.26%, respectively. Since the simulation process only considers the effect of tunnel excavation on the surface settlement, and the actual project also affects the surface settlement of buildings and pipelines on both sides of the tunnel, the maximum settlement value of the curve obtained by fitting the measured data is not in the center of the tunnel, but more to the left. The "three-stage analysis method" proposed in this paper and the Flac3d numerical simulation method adopted can better reflect the real construction situation of the tunnel.

## Conclusions

Based on Peck's theory, this paper deduced the ground settlement formula under the condition of three parallel tunnels, and put forward the "three-stage analysis method" which is suitable for calculating the settlement curve of three parallel tunnels. In this paper, a three-dimensional model of a three-hole parallel shield tunnel was established by using Flac3d software to simulate the tunnel excavation process. In this paper, the effects of tunnel excavation on surface settlement, soil displacement and deformation of tunnel segments were analyzed. The construction sequence was optimized. For three parallel tunnels, the S-shaped excavation method is better.

1. In this paper, an improved "three-stage analysis method" was proposed to calculate the surface settlement of the tunnel. The three-stage analysis method and numerical simulation method proposed in this paper can better reflect the real construction situation of the tunnel. The maximum settlement value obtained by numerical simulation is − 11.8 mm, and the maximum settlement value predicted by the formula of "three-stage analysis" is − 11.66 mm. Compared with the maximum settlement value of − 11.63 mm obtained by fitting the measured data, the errors are 1.46% and 0.25% respectively.
2. In this paper, the optimal excavation sequence of a three-hole tunnel was explored. A three-hole parallel shield tunnel excavation model was established using Flac3d software to simulate its effects on surface settlement, soil deformation and prior tunnel sheeting under four excavation sequences: "right–center–left", "right–left–center", "center–right–left", and the effects of the four excavation sequences of "right–center (reverse)–left" on the surface settlement, soil deformation and prior tunnel segment. For the surface settlement, the final settlement curve is a large "V" shape symmetrical to the center of the center hole. 11.8 mm, 12.69 mm, 11.73 mm and 11.41 mm are the final settlement amounts for the four Cases.
3. The convergence and deformation of the first tunnel segments were investigated in this paper. For the first tunnel segment deformation, the whole shows an oval shape with flat top and bottom and wide ends when the first tunnel is opened. In the later excavation, the degree of convergence of the first tunnel segment remains stable, and the tunnel as a whole is shifted to the left or to the right. The convergence value of S-type excavation was significantly lower than that of other excavation methods.
4. Comprehensive surface and soil displacement and segment convergence offset, it is suggested that the excavation sequence selection in the actual construction process gives priority to the excavation sequence of Case 4, i.e., three tunnels are excavated in S-shape sequentially. It can provide reference for the actual construction.

## Data Availability

The datasets generated and/or analysed during the current study are not publicly available due to the confidentiality re-quirements of the project but are available from the corresponding author on reasonable request.

## References

[1] Peck, R. B. Deep excavations and tunneling in soft ground. in *Proc. 7th ICSMFE, State of the Art Volume* 225–290 (1969).

[2] Zhu C (2017). Control of surface settlement by considering shield tunneling technology. KSCE J. Civ. Eng..

[3] Xiang Q, Su J, Gao Y, Li X, Cheng X (2022). Construction settlement prediction of shield tunnel in soft-soil area. Jordan J. Civ. Eng..

[4] Ma L, Ding L, Luo H (2014). Non-linear description of ground settlement over twin tunnels in soil. Tunn. Undergr. Space Technol..

[5] Kong F, Lu D, Du X, Shen C (2019). Elastic analytical solution of shallow tunnel owing to twin tunnelling based on a unified displacement function. Appl. Math. Model..

[6] Franza A, Viggiani GMB (2021). Role of shear deformability on the response of tunnels and pipelines to single and twin tunneling. J. Geotech. Geoenviron. Eng..

[7] Islam MS, Iskander M (2021). Twin tunnelling induced ground settlements: A review. Tunn. Undergr. Space Technol..

[8] Hu Y (2022). Ground movement induced by triple stacked tunneling with different construction sequences. J. Rock Mech. Geotech. Eng..

[9] Zhang Q (2019). Surface settlement induced by subway tunnel construction based on modified peck formula. Geotech. Geol. Eng..

[10] Song Z, Tian X, Zhang Y (2019). A new modified peck formula for predicting the surface settlement based on stochastic medium theory. Adv. Civ. Eng..

[11] Deng H-S, Fu H-L, Yue S, Huang Z, Zhao Y-Y (2022). Ground loss model for analyzing shield tunneling-induced surface settlement along curve sections. Tunn. Undergr. Space Technol..

[12] Xu Q (2022). Theoretical prediction model for surface settlement caused by the excavation of new tunnels undercrossing existing tunnels based on modified stochastic medium theory. KSCE J. Civ. Eng..

[13] Deng H (2022). Numerical analysis of ground settlement patterns resulting from tunnel excavation in composite strata. Appl. Sci. Basel.

[14] Hasanpour R, Rostami J, Ünver B (2014). 3D finite difference model for simulation of double shield TBM tunneling in squeezing grounds. Tunn. Undergr. Space Technol..

[15] Nematollahi M, Dias D (2019). Three-dimensional numerical simulation of pile-twin tunnels interaction—Case of the Shiraz subway line. Tunn. Undergr. Space Technol..

[16] Tang X, Gan P, Liu W, Zhao Y (2017). Surface settlements induced by tunneling in permeable strata: a case history of Shenzhen Metro. J. Zhejiang Univ. Sci. A.

[17] Liu X (2022). Settlement characteristic due to excavate parallel tunnels in a fill-rock slope: Model test and numerical analysis. Rock Mech. Rock Eng..

[18] Fang Y, Chen Z, Tao L, Cui J, Yan Q (2019). Model tests on longitudinal surface settlement caused by shield tunnelling in sandy soil. Sust. Cities Soc..

[19] Chen R, Zhang P, Wu H, Wang Z, Zhong Z (2019). Prediction of shield tunneling-induced ground settlement using machine learning techniques. Front. Struct. Civ. Eng..

[20] Song Z, Liu S, Jiang M, Yao S (2022). Research on the settlement prediction model of foundation pit based on the improved PSO-SVM model. Sci. Program..

[21] Qiao FS (2021). Settlement prediction of foundation pit excavation based on the GWO-ELM model considering different states of influence. Adv. Civ. Eng..

[22] Zhe W (2018). Field measurement analysis of the influence of double shield tunnel construction on reinforced bridge. Tunn. Undergr. Space Technol..

[23] Ren T (2022). Calculation method for investigating the behavior of ground surface settlement of underpass buildings in TBM double-line tunnels. Sustainability.

[24] Stallebrass, S. E., Grant, R. J. & Taylor, R. N. A finite element study of ground movements measured in centrifuge model tests of tunnels. in *Proceedings of Geotechnical aspects of underground construction in soft ground* vol. **1996**, 595–600 (1996).

[25] Dong, C. Study on calculation method of surface settlement of multi-row parallel pipe jacking. *D. Anhui University of Construction.* (2021) (**in Chinese**)

[26] Zeng, B. Study on ground surface settlement caused by excavation of three-parallel shield tunnel. *J. Shanxi Science & Technology of Communications*. (2022) (**in Chinese**)

